# Supplementary figures and images for: Preparation and Evaluation of Peptides with Potential Antioxidant Activity by Microwave Assisted Enzymatic Hydrolysis of Collagen from Sea Cucumber Acaudina Molpadioides Obtained from Zhejiang Province in China
Source: Mar Drugs. 2019 Mar 15;17(3):169. doi: 10.3390/md17030169 (PMC6471976; doi:10.3390/md17030169)

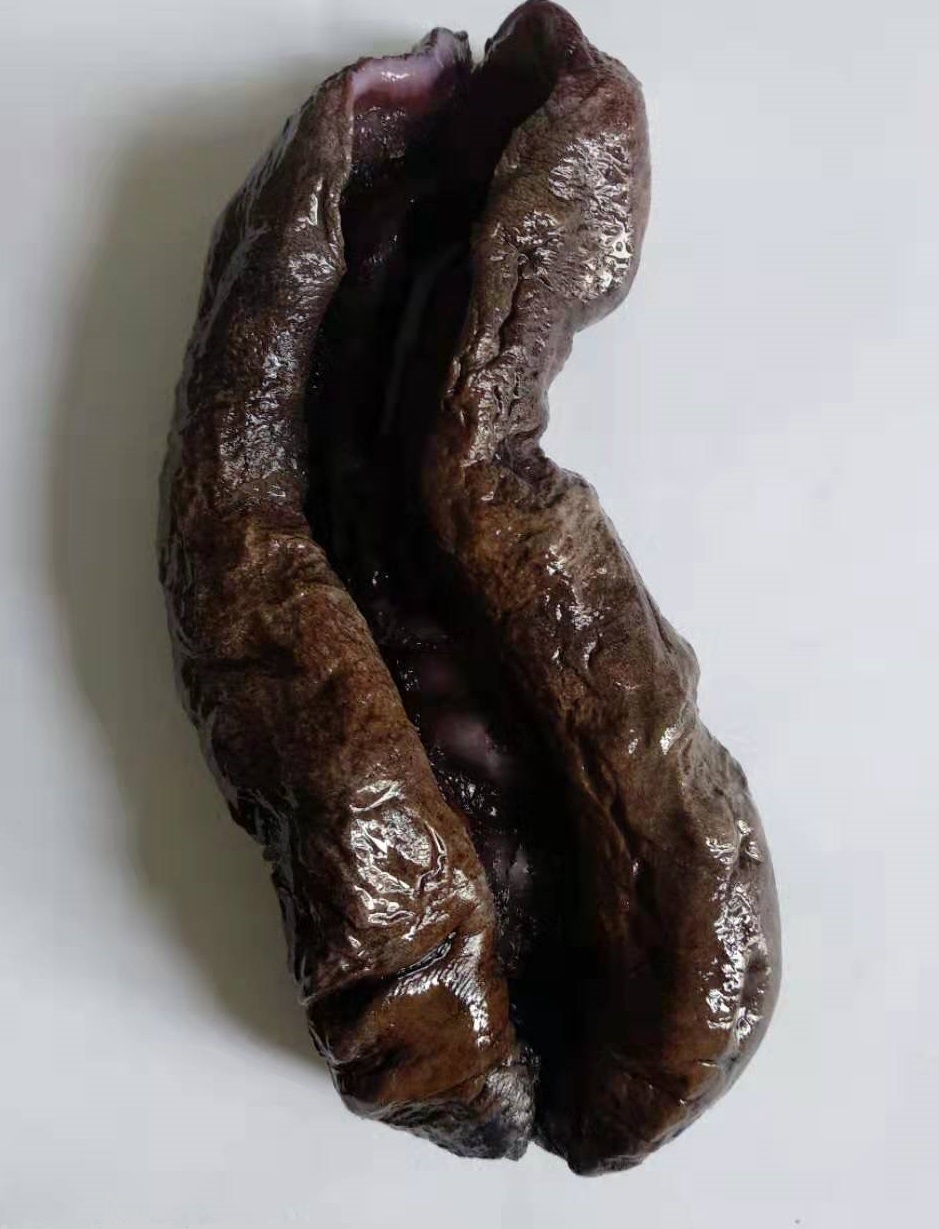

Supplement: Supplementary File 1 [file marinedrugs-17-00169-s001.jpg]
